# CAREGIVER ROLE TRANSITIONS IN HOME-BASED ALZHEIMER’S CARE: A SHANGHAI CASE STUDY

**DOI:** 10.1093/geroni/igae098.3127

**Published:** 2024-12-31

**Authors:** Qingwei Wang, Fengzhi Zhao, Zixuan Wu

**Affiliations:** Xi’an Jiaotong-Liverpool University, Suzhou, Jiangsu, China; Xi’an Jiaotong-Liverpool University, Suzhou, Jiangsu, China (People’s Republic); Johns Hopkins University, Baltimore, Maryland, United States

## Abstract

Alzheimer’s disease poses a growing health crisis in China, exacerbated by the country’s rapidly aging population and cultural emphasis on family-based care. Caregivers shoulder immense physical and psychological burdens, often struggling to reconcile personal expectations with the demands of caregiving. This study, based on ethnographic data collected through prolonged engagements with a family in Shanghai caring for an older adult diagnosed with Alzheimer’s disease, explores the application of Role Theory. This framework explains the sequential stages of role assumption, enactment, ambivalence, and disengagement, offering insights into the complex dynamics of caregiving within China’s cultural context. While existing studies have identified factors influencing caregivers’ “willingness” and “psychological stress” primarily through assessments of psychiatric distress, the practical applications of theoretical models in caregiving practices for this population in China, especially those documented through first-hand experiences, remains underexplored. Drawing from diverse sources, including academic research and firsthand accounts, this study discloses the dynamic process of role transitions in practice, highlighting in particular the transition from a career-focused individual to a full-time caregiver and the subsequent role reversal between parents and children as the illness progresses. By clarifying the dynamic interplay of roles and responsibilities within caregiving relationships, insights gained through this in-depth case study applying Role Theory in practice can offer a roadmap for enhancing caregivers’ well-being and optimizing patient care outcomes.

